# Delayed antibiotic prescribing for respiratory tract infections: individual patient data meta-analysis

**DOI:** 10.1136/bmj.n1282

**Published:** 2021-05-24

In this paper by Stuart and colleagues (*BMJ* 2021;373:n808, doi:10.1136/bmj.n808, published 28 April 2021), the results reported in the abstract for symptom severity should have been “No difference was found in follow-up symptom severity (seven point scale) for delayed versus no antibiotics (adjusted mean difference −0.003, 95% confidence interval −0.12 to 0.11) or delayed versus immediate antibiotics (0.02, −0.11 to 0.15).”

